# Supplementary material for: Rescuing SLAMF3 Expression Restores Sorafenib Response in Hepatocellular Carcinoma Cells through the Induction of Mesenchymal-to-Epithelial Transition
Source: Cancers (Basel). 2022 Feb 12;14(4):910. doi: 10.3390/cancers14040910 (PMC8869973; doi:10.3390/cancers14040910)
Supplement: Supplementary file 1 [file cancers-14-00910-s001.zip › cancers-1577415-supplementary.pdf]

cancers

MDPI

# Supplementary materials: Rescuing SLAMF3 Expression Restores Sorafenib Response in Hepatocellular Carcinoma Cells through the Induction of Mesenchymal-to-Epithelial Transition

**Grégory Fouquet, Constance Marie, Louison Collet, Catherine Vilpoux, Hakim Ouled-Haddou, Eric Nguyen-Khac, Jagadeesh Bayry, Mickaël Naassila, Ingrid Marcq and Hicham Bouhlal**

## Spheroid culture cell assays

To determine sorafenib response, cells were cultured in complete medium and seeded in U-bottomed Corning Ultra Low Attachment 96-wells plates (Dutscher). To obtain the same size spheroids, Ctrl cells were seeded at a density of $2 \times 10^3$ cells/well and Res cells at $4 \times 10^3$ cells/well. Cells were incubated for 4 days at 37 °C in a humidified condition with 5% $CO_2$ prior to treatment.

To analyze the aggressiveness, cells were cultured in complete medium and seeded in flat bottomed Corning Ultra Low Attachment 96-wells plates (Dutscher). Cells were seeded at a density of $4 \times 10^3$ cells/well in complete medium supplemented with 2% Geltrex extracellular matrix (Applied Biosystems, Villebon-sur-Yvette, France). Cells were incubated at 37 °C in a humidified condition with 5% $CO_2$ for five days.

## Cell morphology analysis

To observe cell morphology, cells were cultured for 48 h in complete medium, washed twice with PBS and fixed in methanol for 10 min. Then, cells were immersed with Crystal Violet solution for 5 min, washed with distilled water and air-dried. Cell morphology was quantified using circularity index on imageJ software ranging from 0 to 1. Value 1 corresponded to a round morphology whereas 0 to an elongated cell.

## Cell viability assays and inhibitory concentration 50 calculation (IC50)

The MethylThiazole Tetrazolium (MTT) salt assay was used to evaluate the anti-proliferative effect of sorafenib. After 72 h exposure to different concentration of sorafenib, Ctrl and Res cells, SLAMF3-overexpressing or Mock cells, were rinsed and exposed for 1 h to a 0.5 mg/mL solution of MTT suspended in culture medium. Reduced purple Formazan crystals were extracted with DMSO:Isopropanol (50:50) and analyzed at a wavelength of 570 nm using Tecan Infinite M200 Pro.

To determine sorafenib efficacy in a tridimensional model, spheroids were generated for 4 days and then treated with different concentrations of sorafenib for another 3 days. Seven days after seeding, cell viability was determined using Cell Titer-Glo 3D, according to the manufacturer's protocol (Promega). After transfer to Lumitrac 96-wells plate, luminescence was measured using Tecan Infinite M200 Pro.

The IC50 values of sorafenib were calculated using GraphPad Prism 8 software (GraphPad Software, Inc., La Jolla, CA, USA).

## Clonogenic assay

The inhibitory effect of sorafenib on the proliferation of HCC Ctrl and Res cells was determined by clonogenic assay. Ctrl/Res cells were seeded into 24-well plates at a density of 1,000 cells/well. After 24 h, cells were treated with increasing concentrations of Sorafenib (5 to 20 μM). Two weeks later, colonies were fixed with methanol, stained with crystal violet and photographed. The clonogenicity was analyzed by observing the number of colonies.

**Migration and invasion assays**

For the migration assays, cells were seeded in the upper side of Boyden chambers (BD Biosciences, 24-wells plates, 8 µm pore size) in 200 µL of DMEM 10% FCS. The lower side was filled with 800 µL of DMEM without serum. Cells were incubated for 24 h at 37 °C. Then, cells were washed twice with PBS, fixed with methanol 10 min, stained with crystal violet 5 min and washed thrice in water. Remaining cells in the upper side were removed using a swab. Migrating cells were observed with an inverted microscope. The number of migrating cells was counted in 20 separate fields for three Boyden chambers per condition. For migration analysis, the number of migrating cells of Res condition was normalized by the number of Ctrl migrating cells. Invasion assays were made in the same manner as migration except the upper side of the migration chamber which was coated with Matrigel.

**RNA extraction, quantitative real time PCR**

Total RNA was extracted using ReliaPrep™ RNA Miniprep Systems (Promega, Charbonnières-les-bains, France) and 1 µg of total RNA was reverse transcribed into cDNA with multiscribe reverse transcriptase (Applied Biosystems). Quantitative PCR was performed according to the SYBRGreen Gene Expression protocol (Applied Biosystems) and the expression of HPRT1 was used as an endogenous housekeeping control. For quantitative Real Time PCR (RT-PCR), the following primers were presented in Table S1.

**Western blot analysis**

For the total protein extraction, Ctrl and Res cells were lysed in Nonidet P40 (NP40) buffer (1% NP40, 50 mM Tris pH 7.5, 10% glycerol, 150 mM NaCl, 1 mM EDTA, 100 mM $Na_3VO_6$, 0.5 mM phenylmethanesulphonyl fluoride (PMSF)) containing protease and phosphatase inhibitors (Roche, Meylan, France) and with Triton-X100 at a final concentration of 0.1%.

To discriminate the protein translocation from cytosol to nucleus, a separation of cytoplasmic and nuclear proteins was realized. A first step without Triton-X100 was performed to recover the cytoplasmic fraction. Then, a second step with 0.1% Triton-X100 was executed to recover nuclear proteins. After measuring the protein concentration, equal amounts of each protein sample was separated by electrophoresis, transferred onto PVDF membranes (Bio-Rad, Munich, Germany) and blotted with antibodies presented in Table S2. Blots were developed with the Enhanced ChemiLuminescence (ECL) system (Bio-Rad, Munich, Germany) and analyzed with Image Lab Software (Bio-Rad).

**Immunofluorescence assays**

For immunofluorescence assays, 5,000 Ctrl or Res cells were seeded on slides for 48 h after transfection. After washing twice with PBS (PB 0.1 M NaCl 0.9%, 0.2% Triton-X100, pH 7.5), cells were fixed with pure methanol during 15 min at room temperature. Cells were permeabilized and saturated with donkey and goat blocking serum (PBS 0.1 M; 0.1% Triton-X100; 5% donkey serum, 2% goat serum) for 1 h at room temperature and then incubated with primary antibodies against E-cadherin, vimentin or SLAMF3 (Table S2) overnight at 4 °C. Following extensive washes, cells were incubated with fluorescent-conjugated secondary antibodies for 1 h at room temperature (Table S2). Cells were washed twice with PBS, incubated with 50 µg/mL of DAPI during 15 min. Following washes in PBS, slides were mounted on lamellae with mounting solution (Life Technologies Prolong Gold P36934). The specificity of the two antibodies was tested by omitting the primary antibody from an additional slide. In such conditions, no immunoreactive cells were detected. The cells were examined and photographed under a fluorescence microscope at X40 magnification (Olympus).

**Flow cytometry assays**

Cells were collected in cold PBS/2.5% mice serum, washed, saturated for 20 min in PBS/2.5% serum and then incubated with fluorescent-conjugated primary antibodies presented in Table S2 or isotype-matched antibodies for 20 min at 4 °C. Following extensive wash with PBS, 50,000 viable events were analyzed (MACSQuant cytometer running MACSQuantify software; Miltenyi Biotec, Paris, France). Results were expressed as percentage of positive cells and the MFI ratio (the MFI obtained in the presence of specific antibody divided by the MFI obtained with a non-specific, matched isotype). Data were analyzed using the FlowJo software (FlowJo LLC).

**Adipogenic differentiation**

For adipogenic differentiation, 15 × $10^3$ cells (Ctrl, Res and MSC) were seeded into six-well plates for 24 h. Then, culture medium was removed and replaced with adipogenic medium (DMEM Low glucose, 10% FCS, 2 mM L-glutamine, 100 UI/mL Penicillin, 100 µg/mL Streptomycin, 500 µM Isobutylmethylxanthine, 60 µM Indomethacin and 1 µM Dexamethasone). Adipogenic medium was changed twice a week. After 7 and 14 days differentiation, the intracellular lipid accumulation was assessed using Oil-Red O staining according to the manufacturer's protocol (Sigma-Aldrich). The level of induction of adipogenesis-related genes (PPARγ, C/EBPα, GLUT4, Leptin and FABP4) was determined using RTqPCR.

**Cell death assay**

For the apoptosis assay, the Annexin V-FITC (AV)/Propidium iodide (PI) kit (Invitrogen) was used according to the manufacturer's instructions 24 h after exposition of Ctrl and Res cells to DMSO, 5 µM or 10 µM Sorafenib. Cell death assay was analyzed by flow cytometry (MACSQuant cytometer running MACSQuantify software; Miltenyi Biotec, Paris, France) and was analyzed using the FlowJo software (FlowJo LLC).

**Cell size and complexity analysis**

Ctrl and Res cells morphology was analyzed by flow cytometry using MACSQuant (Miltenyi Biotec). Results are presented as cell cytograms indicating size (FSC) and complexity or granularity (SSC) of Ctrl and Res cells.

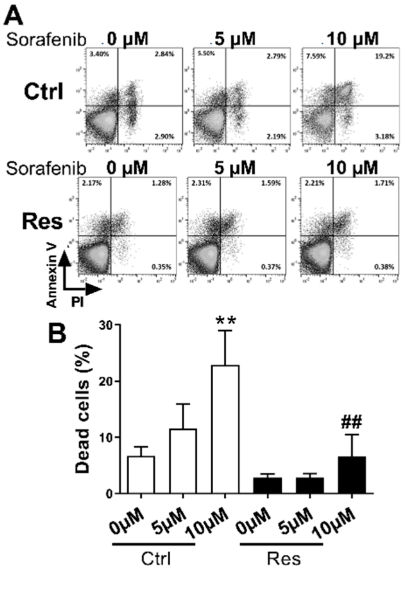


**Figure S1.** Res cells are resistant to death-induced by sorafenib. (**A** and **B**) Induction of cell death by sorafenib as determined by flow cytometry in Ctrl and Res cells. (**A**) Representative dot plots and

(B) quantification of dead cells form six independent experiments (** $p < 0.01$ vs respective 0 µM; ## $p < 0.01$ vs respective Ctrl condition).

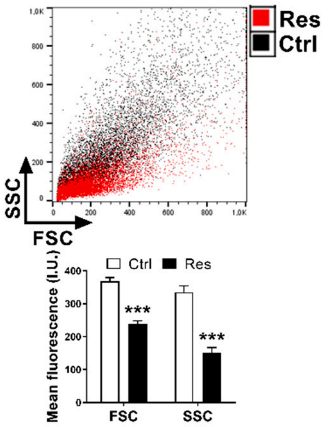


**Figure S2.** Long-term exposure to sorafenib induces modification of cell morphology associated with EMT. Flow cytometry dot plots of forward and side scatter (FSC-SSC) in Ctrl (black) and Res (red) cells. Results are presented as mean ± SEM (*** $p < 0.001$ vs respective Ctrl condition).

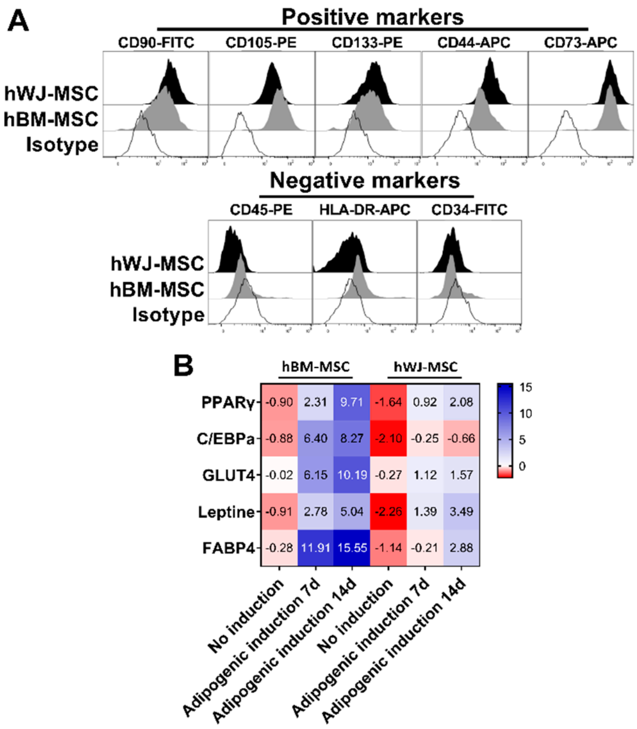


**Figure S3.** Adipogenic differentiation of human Mesenchymal Stem Cells. Human MSC were exposed to adipogenic differentiation medium for 7 or 14 days. (**A**) Analyses of MSC positive markers CD90, CD105, CD133, CD44 and CD73, and MSC negative markers CD45, HLA-DR and CD34 by flow cytometry. (**B**) Heat map representing the fold changes in the mRNA expression of adipogenic-related genes under various culture conditions.

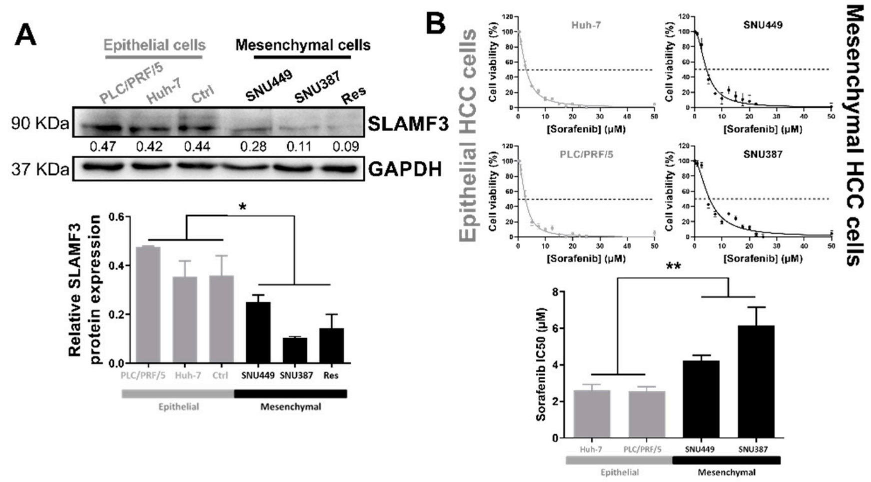


**Figure S4.** SLAMF3 expression and sorafenib response in different HCC cell lines. (**A**) SLAMF3 expression was analyzed in the four HCC cell lines by western blot. Representative blots and densitometry analysis of western blots from three experiments are presented (* $p < 0.05$ Epithelial cell lines vs Mesenchymal cell lines). (B) Two epithelial HCC cell lines Huh-7, PLC/PRF/5, and two mesenchymal HCC cell lines SNU449 and SNU387 were cultured with 0–50 μM sorafenib and MTT assay was performed 72 h after treatment. Results are represented as mean ± SEM of four independent experiments (** $p < 0.01$ Epithelial cell lines vs Mesenchymal cell lines).

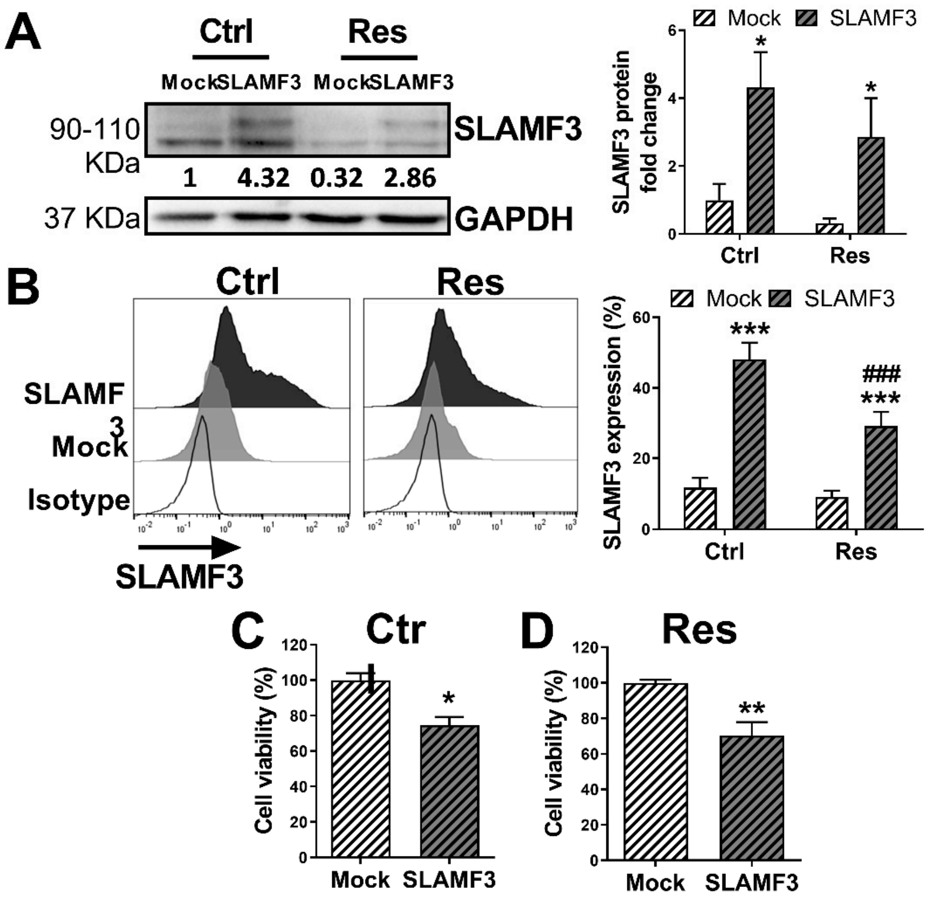


**Figure S5.** SLAMF3 overexpression decreases the cell viability in Ctrl and Res HCC cells. Cells were transfected with SLAMF3 plasmid (SLAMF3) or empty plasmid (Mock) and cultured for 48 h. (**A**)

Analysis of expression of SLAMF3 by western blot. Representative plot images and densitometry analysis of western blots from six independent experiments (*N* = 6; * $p < 0.05$ *vs* respective Mock condition, densitometry ratio of each band of western blots are mentioned under bands). (**B**) SLAMF3 expression analysis in Mock and SLAMF3-transfected conditions by flow cytometry. Results represent one of the five independent experiments (*N* = 5) and graphs represent mean ± SEM of SLAMF3 positive cells (*N* = 5, *** $p < 0.001$ vs respective Mock condition and ### $p < 0.001$ vs respective Ctrl condition). (**C** and **D**) Effect of SLAMF3 overexpression on cell viability as determined by MTT assay in (**C**) Ctrl and (**D**) Res cells, respectively 96 h after transfection (*N* = 4; * $p < 0.05$ vs Mock condition).

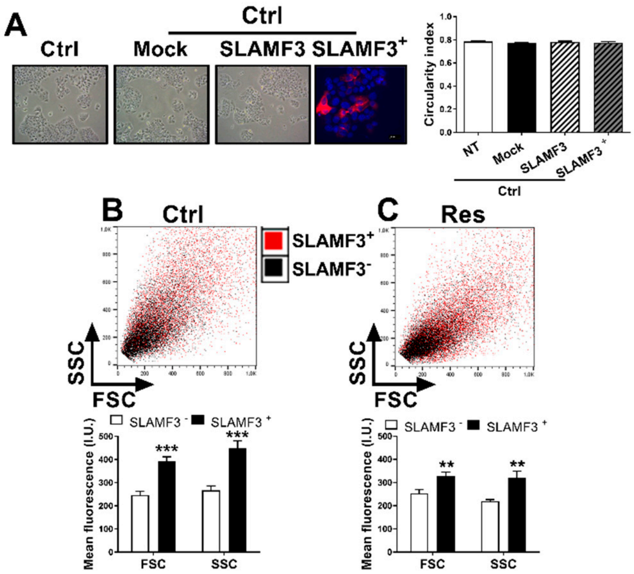


**Figure S6.** SLAMF3 overexpression modifies HCC cell morphology. (**A**) Images of Ctrl cells in culture conditions or immunofluorescence staining for SLAMF3. Circularity index was analyzed by ImageJ software (*N* = 3; *n* = 138 for Ctrl cells, *n* = 86 for Ctrl SLAMF3+). (**B** and **C**) Flow cytometry dot plots of forward and side scatter (FSC-SSC) under Mock (black) and SLAMF3 (red) transfected conditions in Ctrl (**B**) and Res (**C**) cells. Results are presented as mean ± SEM (** $p < 0.01$ and *** $p < 0.001$ vs respective Ctrl condition).

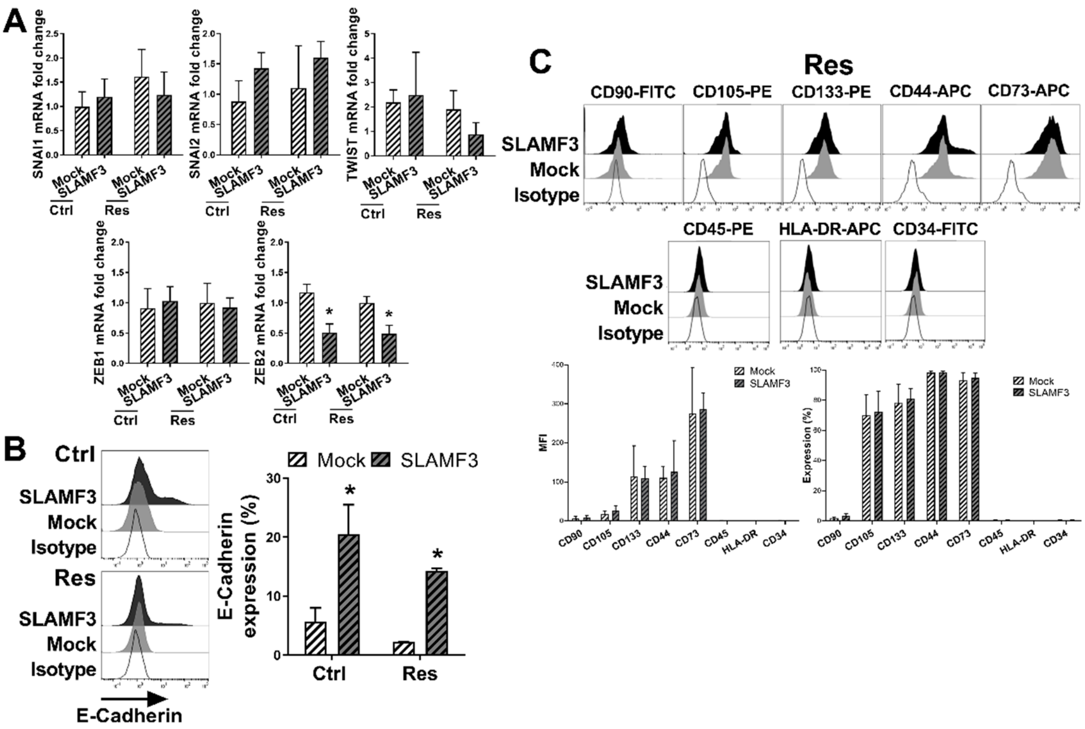


**Figure S7.** Analysis of expression of EMT markers in HCC cells overexpressing SLAMF3. (**A**) Quantitative RT-PCR analysis of expression of *SNAI1*, *SNAI2*, *Twist*, *Zeb1* and *Zeb2* in transfected cells. (**B**) Flow cytometry-based analysis of E-cadherin protein in Ctrl and Res cells transfected with SLAMF3 plasmid (SLAMF3) or empty plasmid (Mock). Representative plot overlays and mean ± SEM from the four independent experiments (*** $p < 0.001$ vs respective SLAMF3$^-$ cells). (**C**) Analyses of MSC positive markers CD90, CD105, CD133, CD44 and CD73, and MSC negative markers CD45, HLA-DR and CD34 by flow cytometry in Mock and SLAMF3-transfected Res cells. Representative plot overlays and mean ± SEM (N=3) of mean fluorescence intensity (MFI) or percentage positive are presented.

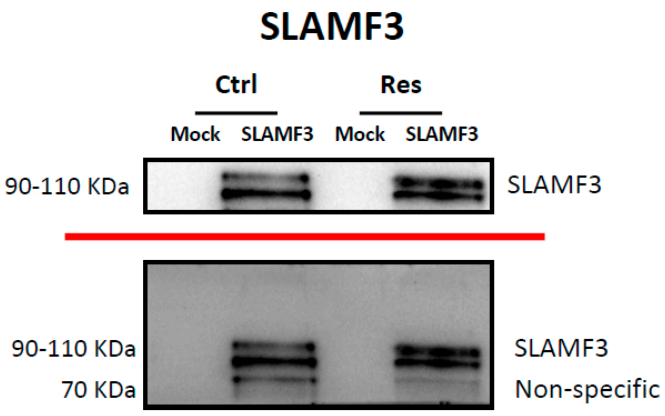

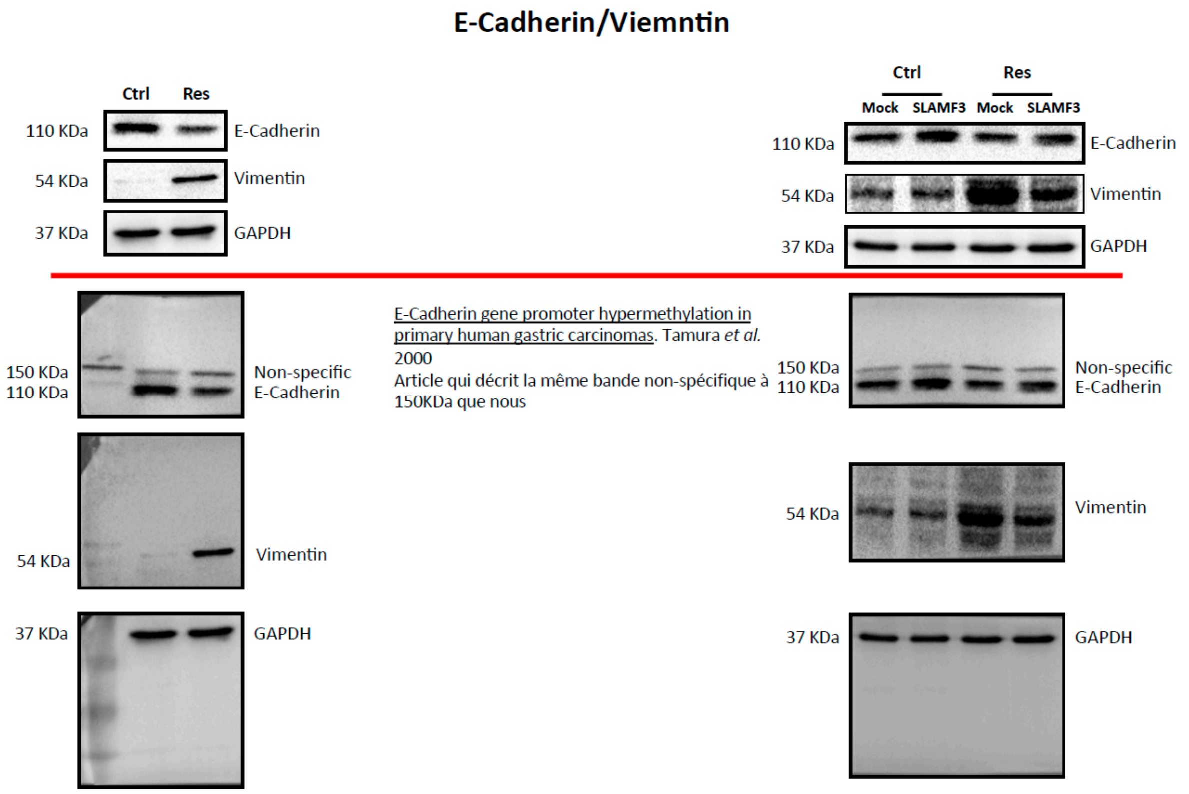
E-Cadherin/Viemntin
Ctrl
Res
110 KDa
E-Cadherin
54 KDa
Vimentin
37 KDa
GAPDH
Mock
SLAMF3
Mock
SLAMF3
150 KDa
110 KDa
Non-specific
E-Cadherin
E-Cadherin gene promoter hypermethylation in primary human gastric carcinomas. Tamura et al. 2000
Article qui décrit la même bande non-spécifique à 150KDa que nous
54 KDa
Vimentin
37 KDa
GAPDH

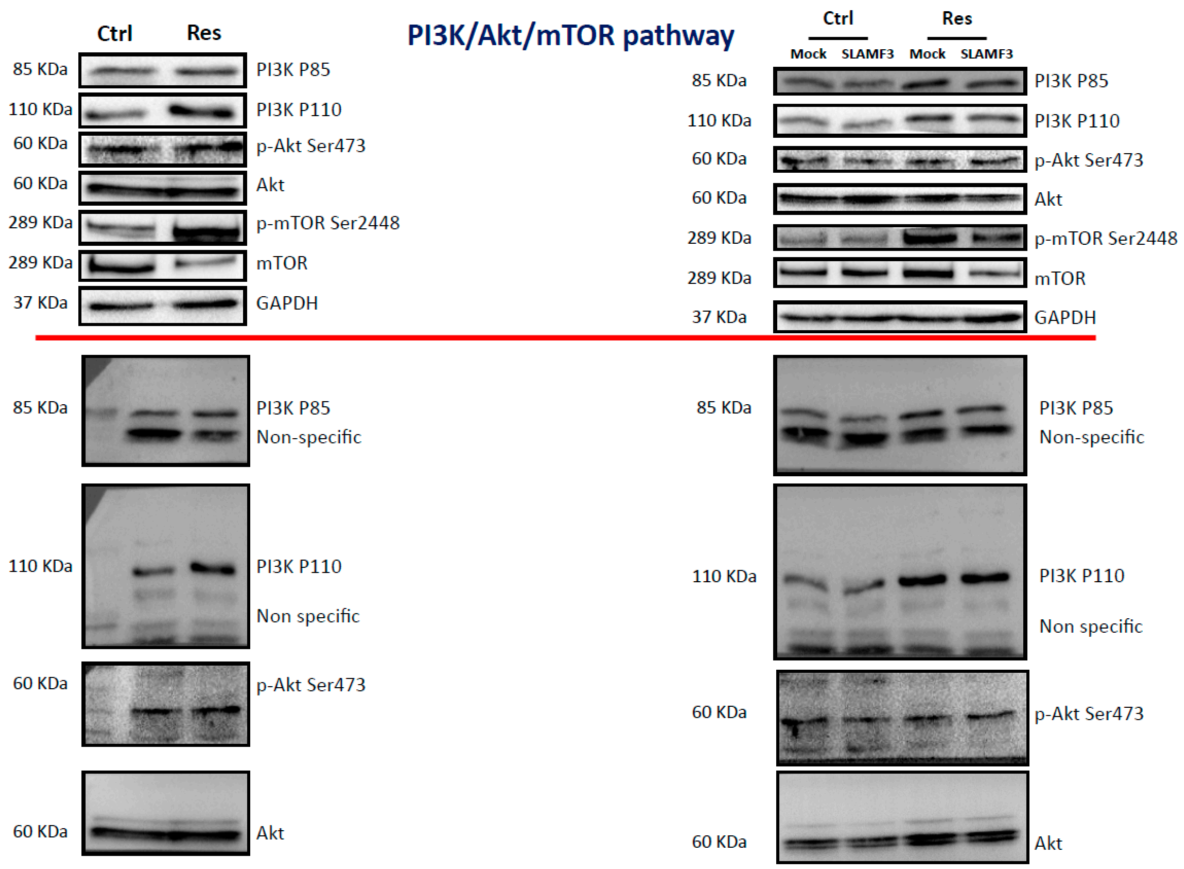
PI3K/Akt/mTOR pathway
Ctrl
Res
Mock
SLAMF3
Mock
SLAMF3
85 KDa
PI3K P85
110 KDa
PI3K P110
60 KDa
p-Akt Ser473
60 KDa
Akt
289 KDa
p-mTOR Ser2448
289 KDa
mTOR
37 KDa
GAPDH
85 KDa
PI3K P85
Non-specific
110 KDa
PI3K P110
Non specific
60 KDa
p-Akt Ser473
60 KDa
Akt

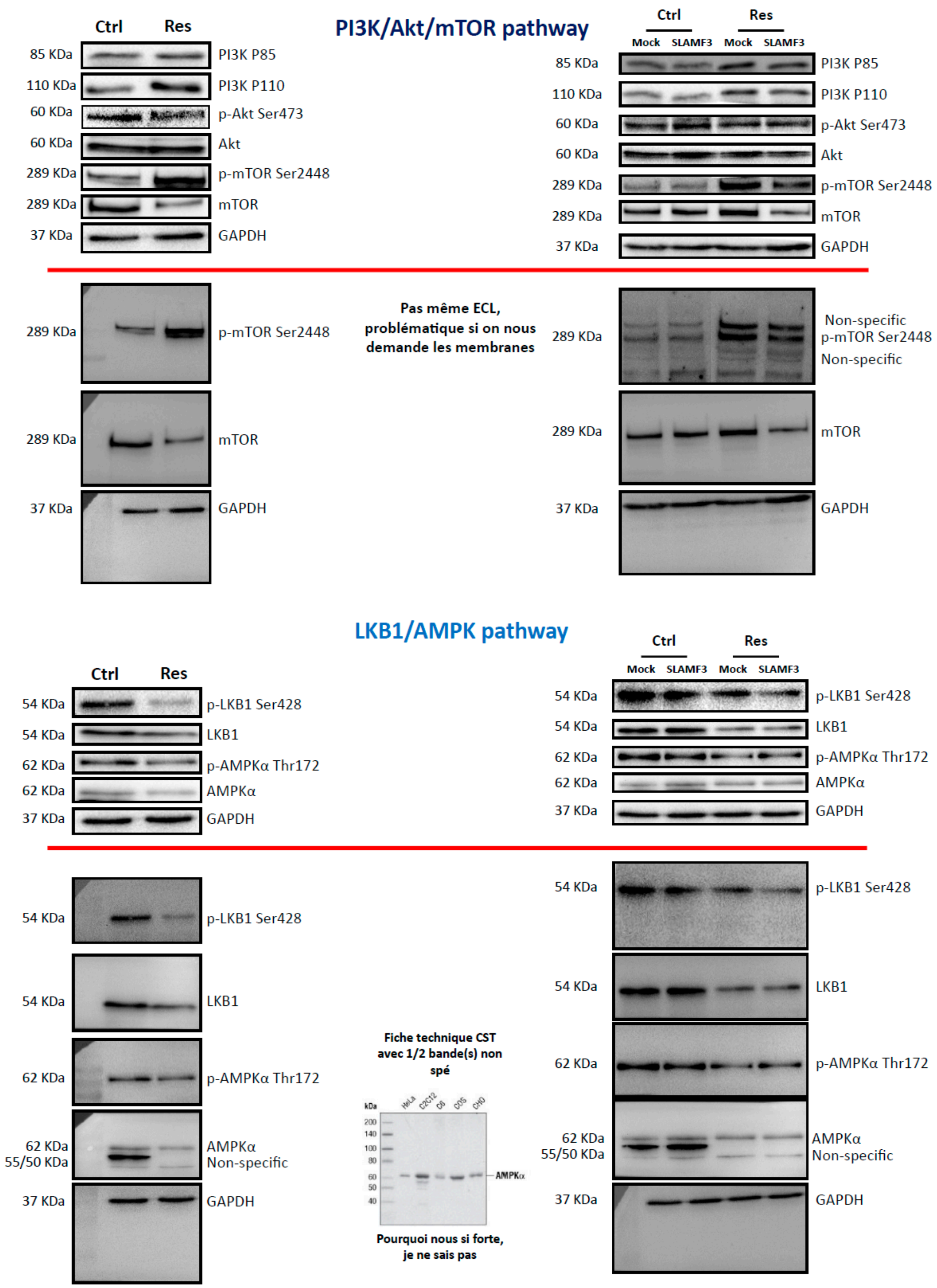
PI3K/Akt/mTOR pathway
Ctrl
Res
Mock
SLAMF3
85 KDa
110 KDa
60 KDa
289 KDa
37 KDa
PI3K P85
PI3K P110
p-Akt Ser473
Akt
p-mTOR Ser2448
mTOR
GAPDH
Pas même ECL, problématique si on nous demande les membranes
Non-specific
LKB1/AMPK pathway
54 KDa
62 KDa
55/50 KDa
p-LKB1 Ser428
LKB1
p-AMPKα Thr172
AMPKα
Fiche technique CST avec 1/2 bande(s) non spé
kDa
HeLa
C2C12
C6
COS
CHO
200
140
100
80
60
50
40
Pourquoi nous si forte, je ne sais pas

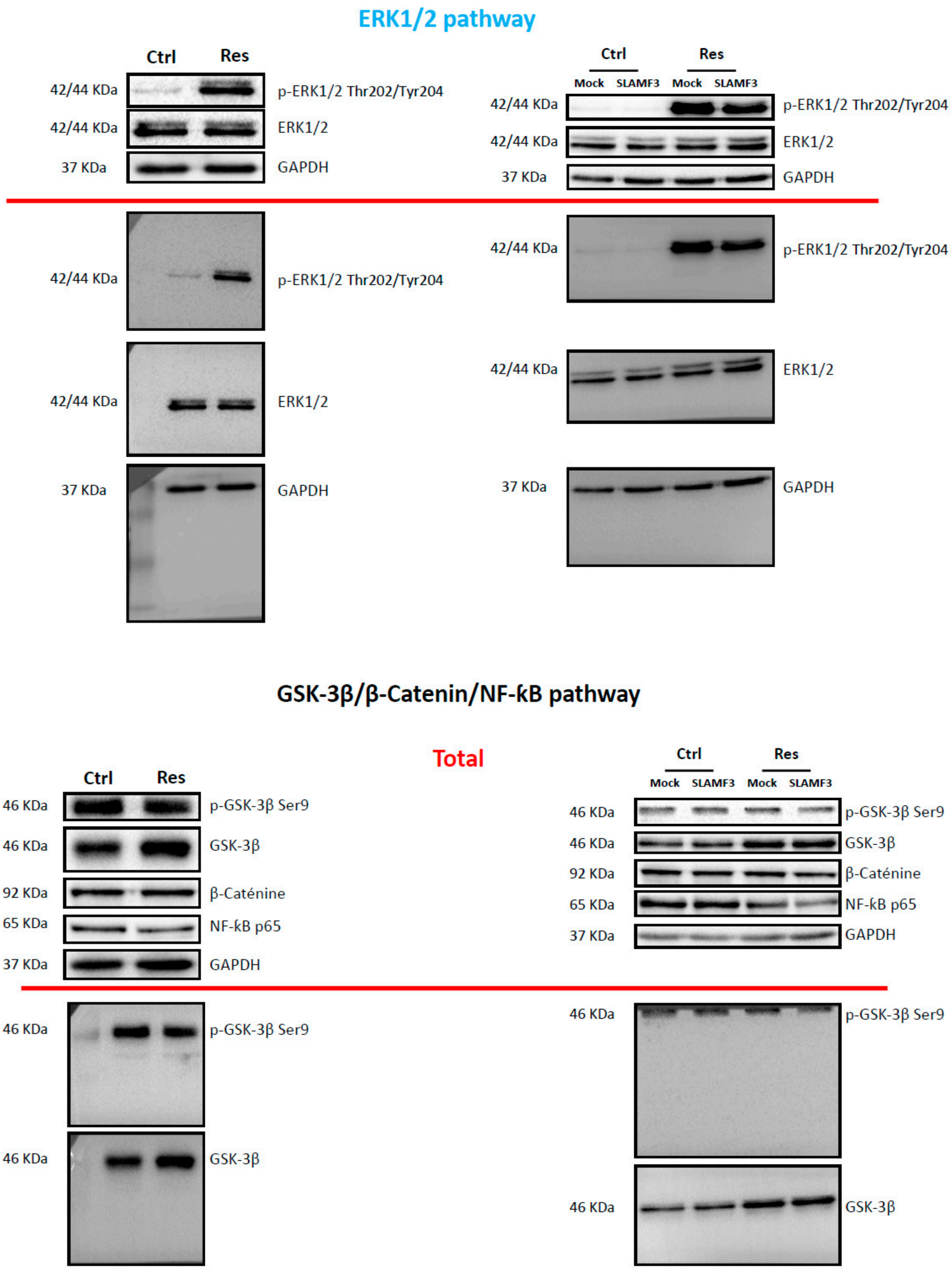
ERK1/2 pathway
Ctrl
Res
Mock
SLAMF3
42/44 KDa
p-ERK1/2 Thr202/Tyr204
ERK1/2
37 KDa
GAPDH
GSK-3β/β-Catenin/NF-kB pathway
Total
46 KDa
p-GSK-3β Ser9
GSK-3β
92 KDa
β-Caténine
65 KDa
NF-kB p65
37 KDa
GAPDH

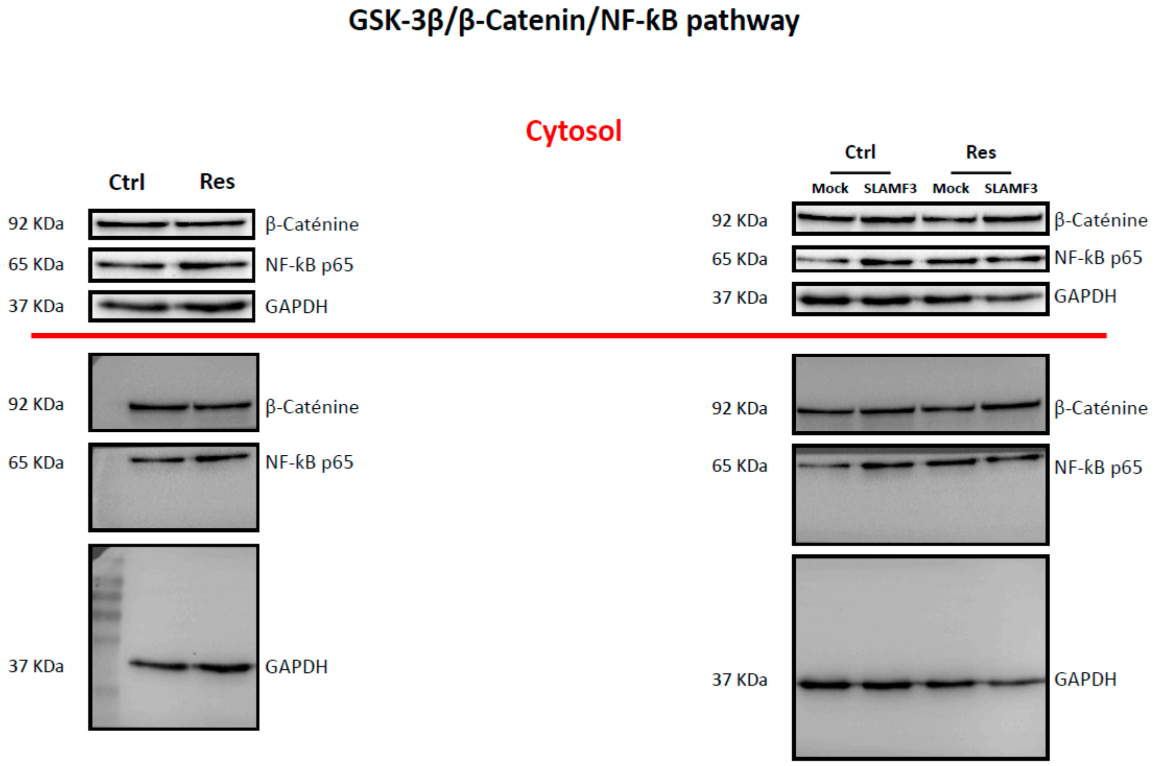
GSK-3β/β-Catenin/NF-kB pathway
Cytosol
Ctrl
Res
Mock
SLAMF3
Mock
SLAMF3
92 KDa
65 KDa
37 KDa
β-Caténine
NF-kB p65
GAPDH

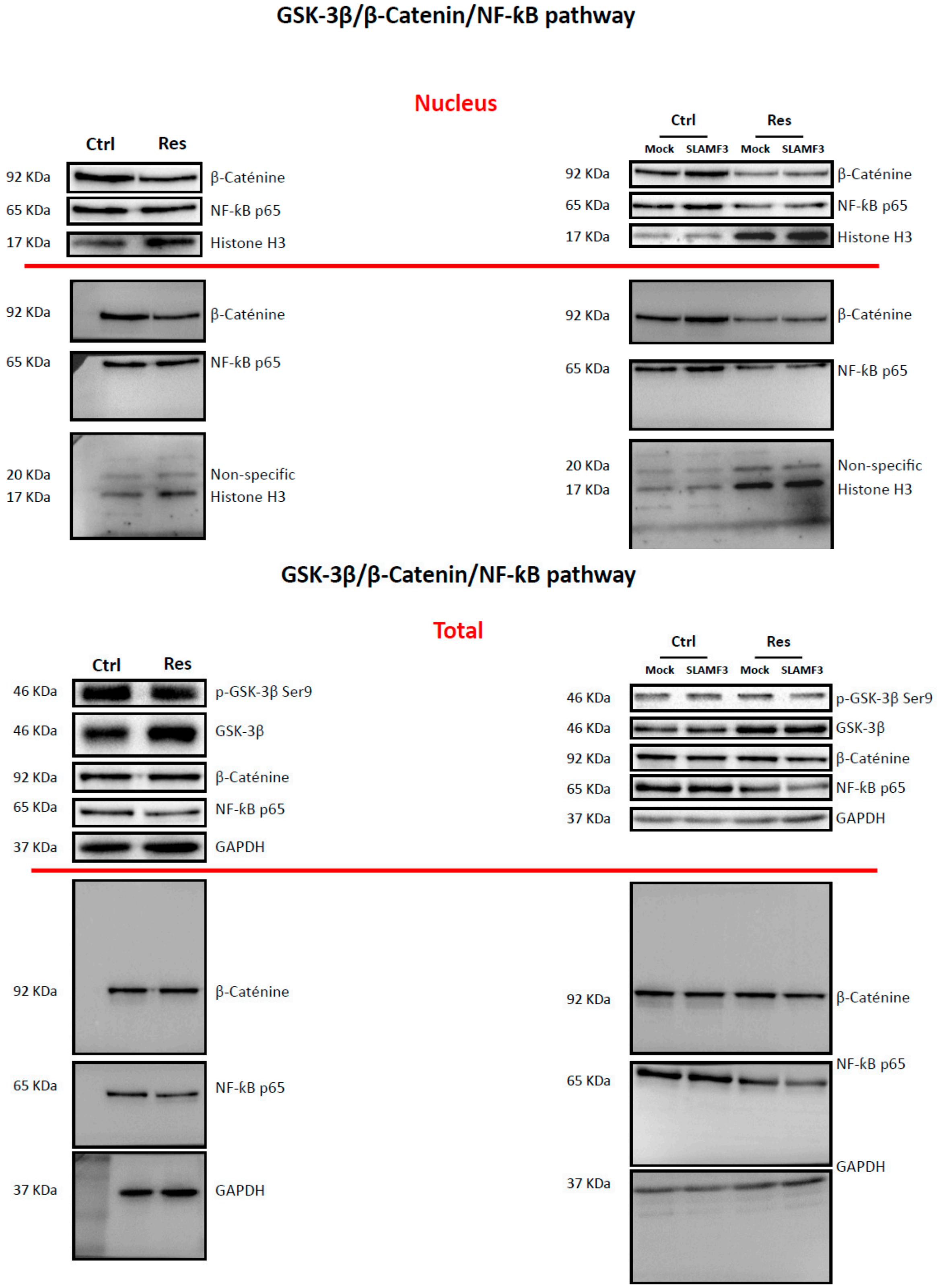


**Figure S8.** Full pictures of the Western blots.

**Table S1.** Primers for SYBRGreen quantitative RT-PCR.

| | Forward | Reverse |
|---|---|---|
| CDH1 | 5'- CGAGAGCTACACGTTCACGG -3' | 5'- CACACCATCTGTGCCCACTT -3' |
| VIM | 5'- GCACGTCTTGACCTTGAACG -3' | 5'- GGTCAGGCTTGGAAACATCC -3' |

| | | |
|---|---|---|
| SNAI1 | 5'- GACCCCAATCGGAAGCCTAA -3' | 5'- AGATGAGCATTGGCAGCGA -3' |
| SNAI2 | 5'- ACAGCGAACTGGACACACAT -3' | 5'- GCGGTAGTCCACACAGTGAT -3' |
| TWIST | 5'- CAGTCGCTGAACGAGGCG -3' | 5'- CTGCAGCTTGCCATCTTGGA -3' |
| ZEB1 | 5'- TACCAGAGGATGACCTGCCA -3' | 5'- CTTCAGGCCCCAGGATTTCT -3' |
| ZEB2 | 5'- CGCAAACAAGCCAATCCCAG -3' | 5'- TCTCCTGGTCCAGAGGGTTG -3' |
| PPARγ | 5'- GACCACTCCCACTCCTTTGA -3' | 5'- AGATGCAGGCTCCACTTTGA -3' |
| C/EBPα | 5'- GGTGGACAAGAACAGCAACG -3' | 5'- GCGGTCATTGTCACTGGTCA -3' |
| GLUT4 | 5'- CTGTGTTCTCTGCGGTGCTT -3' | 5'- AGCCACGTCTCATTGTAGCTC -3' |
| FABP4 | 5'- TGGTTGATTTTCCATCCCAT -3' | 5'- TACTGGGCCAGGAATTTGAC -3' |
| LEP | 5'- TCTATGTCCAAGCTGTGCCC -3' | 5'- GAGCCCAGGAATGAAGTCCA -3' |
| HPRT1 | 5`- TGACCTTGATTTATTTTGCATACC -3' | 5`- CGAGCAAGACGTTCAGTCCT -3' |

**Table S2.** List of antibodies used in this study.

| **Immunofluorescence assay** | | |
|---|---|---|
| **Target protein** | **Manufacturers** | **Dilution** |
| E-cadherin | Abcam | 1/50 |
| Vimentin | Abcam | 1/250 |
| SLAMF3-Biotin | Miltenyi Biotec | 1/50 |
| Anti-rabbit AlexaFluor 647 | Abcam | 1/250 |
| Anti-mouse AlexaFluor 488 | Abcam | 1/250 |
| Streptavidine-TRITC | Jackson ImmunoResearch | 1/250 |
| **Flow cytometry assays** | | |
| **Target protein** | **Manufacturers** | **Dilution** |
| CD90-FITC | BD Biosciences | 1/50 |
| CD105-PE | BD Biosciences | 1/50 |
| CD133-PE | BD Biosciences | 1/50 |
| CD44-APC | BD Biosciences | 1/50 |
| CD73-APC | BD Biosciences | 1/50 |
| CD45-PE | BD Biosciences | 1/50 |
| HLA-DR-APC | BD Biosciences | 1/50 |
| CD34-FITC | BD Biosciences | 1/50 |
| SLAMF3-PE-Vio770 | Miltenyi-Biotec | 1/50 |
| E-cadherin | Abcam | 1/50 |
| Anti-mouse AlexaFluor 488 | Abcam | 1/250 |
| **Western blot analysis** | | |
| **Target protein** | **Manufacturers** | **Dilution** |
| E-cadherin | Abcam | 1/500 |
| Vimentin | Abcam | 1/1000 |
| PI3K P85 | Cell Signaling Technology | 1/1000 |
| PI3K P110 | Cell Signaling Technology | 1/1000 |
| p-Akt Ser473 | Cell Signaling Technology | 1/200 |
| Akt | Cell Signaling Technology | 1/1000 |
| p-mTOR Ser2448 | Cell Signaling Technology | 1/1000 |
| mTOR | Cell Signaling Technology | 1/1000 |
| p-GSK-3β Ser9 | Cell Signaling Technology | 1/1000 |
| GSK-3β | Cell Signaling Technology | 1/1000 |
| β-catenin | Cell Signaling Technology | 1/1000 |
| p-ERK1/2 Thr202/Tyr204 | Cell Signaling Technology | 1/1000 |
| ERK1/2 | Cell Signaling Technology | 1/1000 |
| NF-ĸB | Cell Signaling Technology | 1/1000 |
| p-LKB1 Ser428 | Cell Signaling Technology | 1/1000 |
| LKB1 | Cell Signaling Technology | 1/1000 |
| p-AMPKα Thr172 | Cell Signaling Technology | 1/1000 |
| AMPKα | Cell Signaling Technology | 1/1000 |
| SLAMF3 (CD229) | Santa Cruz Biotechnology | 1/200 |
| GAPDH | Clinisciences | 1/1000 |

| | | |
|---|---|---|
| Histone H3 | Upstate Cell Signaling Solution | 1/500 |
